# The interplay between nutrigenomics and low-carbohydrate ketogenic diets in personalized healthcare

**DOI:** 10.3389/fnut.2025.1595316

**Published:** 2025-06-23

**Authors:** Yousef M. Almoghrabi, Basmah M. Eldakhakhny, Abdulhadi I. Bima, Hussein Sakr, Ghada M. A. Ajabnoor, Hoda M. Gad, Fatma Azzahraa H. Mohammad, Salma A. Elsamanoudy, Akram Z. Awan, Ayman Z. Elsamanoudy

**Affiliations:** ^1^Clinical Biochemistry Department, Faculty of Medicine, King Abdulaziz University, Jeddah, Saudi Arabia; ^2^King Fahd Medical Research Center, Regenerative Medicine Unit, King Abdulaziz University, Jeddah, Saudi Arabia; ^3^Food, Nutrition, and Lifestyle Research Unit, King Fahd for Medical Research Centre, King Abdulaziz University, Jeddah, Saudi Arabia; ^4^Department of Physiology, College of Medicine and Health Sciences, Sultan Qaboos University, Muscat, Oman; ^5^Department of Medical Physiology, Faculty of Medicine, Mansoura University, Mansoura, Egypt; ^6^Medical Biochemistry and Molecular Biology Department, Faculty of Medicine, Alexandria University, Alexandria, Egypt; ^7^Medical Biochemistry and Molecular Biology Department, Faculty of Medicine, Mansoura University, Mansoura, Egypt; ^8^Mansoura-Manchester Medical Program for Medical Education, Faculty of Medicine, Mansoura University, Mansoura, Egypt; ^9^Faculty of Medicine, King Abdulaziz University, Jeddah, Saudi Arabia

**Keywords:** nutrigenomics, nutrigenetics, ketogenic diet, low-carbohydrate, personalized healthcare

## Abstract

The field of nutrigenomics explores the interaction between diet and gene expression, examining how nutrients function as signaling molecules that influence cellular processes, protein synthesis, and metabolite production. This discipline aims to design diets that promote genomic stability through various genetic mechanisms, including minimizing DNA damage and epigenetic modifications, among others. Nutrigenomic profiling helps identify individual dietary needs and responses to food interventions. Nutrigenetics, a subfield of nutritional genomics, investigates how genetic variations affect nutrient metabolism and health outcomes. It operates on the principle that genetic differences influence nutrient absorption and metabolism, shaping disease susceptibility and treatment responses. Therefore, integrating nutrigenetics into dietary planning enables the development of personalized nutrition strategies to improve health and prevent disease. One key application of nutrigenomics is its impact on various diets, including the Low-Carbohydrate ketogenic diet (LCKD), a high-fat, low-carbohydrate regimen that induces ketosis. In this metabolic state, ketone bodies serve as energy sources and signaling molecules. Research suggests that LCKD may influence gene expression and epigenetic mechanisms, modulating inflammation-related pathways, oxidative stress, and metabolic regulation. Additionally, KD has been associated with improved insulin sensitivity, glucose control, lipid profiles, and weight loss. However, genetic factors affecting LCKD response require further investigation to refine personalized dietary recommendations. This review highlights the significance of nutrigenomics, focusing on the interplay between the LCKD and genetic factors. A comprehensive understanding of these interactions is essential for developing personalized dietary strategies that optimize LCKD benefits while mitigating potential risks, ultimately contributing to individualized nutritional guidance within a precision health framework.

## 1 Introduction

Nutritional genomics is an emerging interdisciplinary field that explores how dietary components interact with the genome to influence health and disease. It encompasses two complementary domains: nutrigenomics, which investigates how nutrients and other dietary compounds influence gene expression and regulation (1–3), and nutrigenetics, which examines how genetic variability affects individual responses to nutrient intake, absorption, and metabolism (1, 2). Nutrients act as signaling molecules, transmitting and interpreting dietary signals within cells that alter gene expression in the nucleus, ultimately leading to changes in protein and metabolite expression (3). By uncovering these mechanisms, nutrigenomic profiling can help explain inter-individual differences in dietary requirements and responses to nutritional interventions (4). This knowledge supports the development of dietary strategies aimed at maintaining genomic stability by minimizing DNA damage, epigenetic changes, and disruptions in transcriptomic, proteomic, and metabolomic profiles (5). Integrating nutrigenetics into personalized dietary planning also has the potential to improve health outcomes and support disease prevention (6). Collectively, these approaches fall under the umbrella of nutritional genomics, offering a holistic view of how food influences gene expression, metabolic regulation, and physiological responses (7). Understanding these gene-diet interactions is especially critical in light of the genomic diversity across populations, differences in nutrient bioavailability, and the role of cultural, geographic, and economic factors in shaping diet and nutrient access (8, 9).

**Figure 1:**
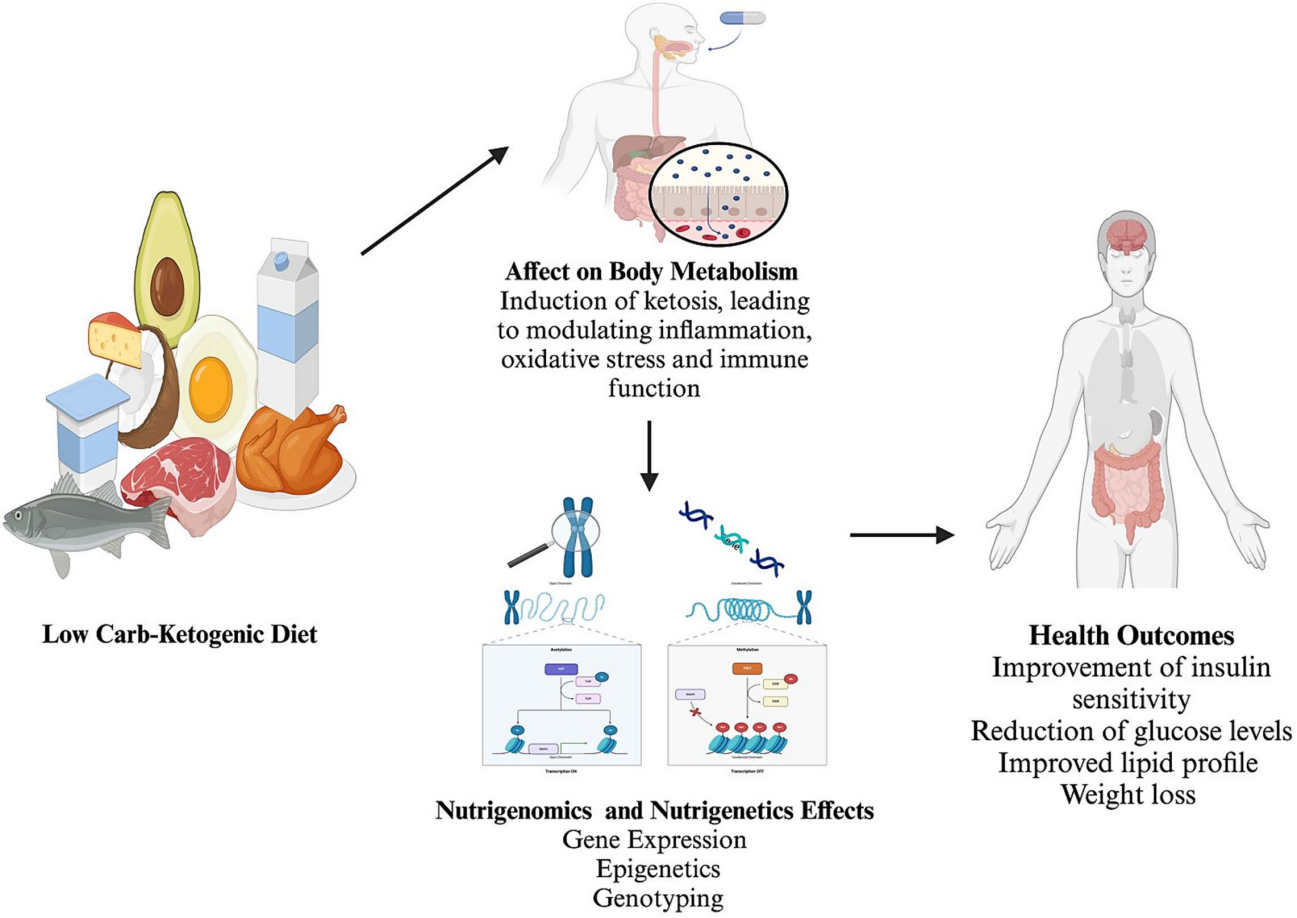
Summary of the effect of the low carbohydrates-ketogenic diet on body metabolism and nutrigenomics with nutrigenetics changes and effects. Created with BioRender.com Ajabnoor, https://BioRender.com/p72p958.

The Low-Carbohydrate ketogenic diet (LCKD) regimen was initially used to manage epilepsy and diabetes before the advent of insulin and antiseizure medications (10). The LCKD induces a state of ketosis, a metabolic state where fats are primarily used as energy sources, producing ketone bodies (11). Ketones are regarded as both metabolic fuels and signaling molecules, modulating gene expression and pathways related to inflammation, oxidative stress, immune function, and cellular health (6). This dietary intervention has been shown to impact metabolic health, including improvements in insulin sensitivity, reductions in blood glucose levels, and favorable changes in lipid profiles (Figure 1). These metabolic changes are also associated with weight loss and reduced low-grade inflammation (12). Furthermore, the LCKD influences epigenetic mechanisms, such as histone deacetylation, which can alter gene expression by modifying DNA and histone proteins (13–15).

However, the genetic variants predictive of LCKD response require further evaluation in intervention trials to avoid misleading individuals and healthcare practitioners (16, 17). Implementing a personalized approach to the ketogenic diet emphasizes its therapeutic potential and long-term safety.

The significance of this review lies in its potential to contribute to the field of personalized medicine. By understanding the genetic bases of diet response, healthcare providers can offer more precise nutritional guidance tailored to individual genetic profiles. This understanding enhances the effectiveness of dietary interventions, such as the ketogenic diet, and improves patient outcomes in various metabolic, neurological, and cardiovascular conditions.

This review aims to explore the intricate interactions between the ketogenic diet, nutrigenetics, and nutrition-related genes, focusing on how a low-carbohydrate, high-fat diet influences gene expression, epigenetics, and genotyping.

## 2 Methods

This scoping review explores the interplay between the ketogenic diet, nutrigenetics, and nutrition-related genes. We aimed to discuss how a low-carbohydrate, high-fat diet influences gene expression, epigenetics, and genotyping and to assess the impact of genetic diversity on the outcomes of adopting a ketogenic diet. A comprehensive search was conducted across databases, including PubMed, Scopus, Web of Science, and Google Scholar, to achieve these targets. The literature search focused on articles published within the last 15 years (2010–2024) to ensure the inclusion of the most current data.

The search employed a combination of keywords and MeSH terms, using phrases such as “ketogenic diet” AND “nutrigenetics,” “low-carbohydrate high-fat diet” AND “gene expression,” “epigenetics” AND “ketogenic diet,” “genetic diversity” AND “ketogenic diet outcomes,” and “genotyping” AND “ketogenic diet.” The inclusion criteria for studies are publication in peer-reviewed journals within the specified 10-year range, focus on the impacts of the ketogenic diet on gene expression, epigenetics, and genetic outcomes, investigation into the role of genetic diversity in the efficacy and outcomes of the ketogenic diet, studies conducted on human subjects, and availability in English. Conversely, exclusion criteria include non-peer-reviewed publications, studies focusing solely on animal models with no clinical implications, research not directly related to the interplay of ketogenic diet and genetics, publications before 2014, and studies not available in full text.

**Figure 2:**
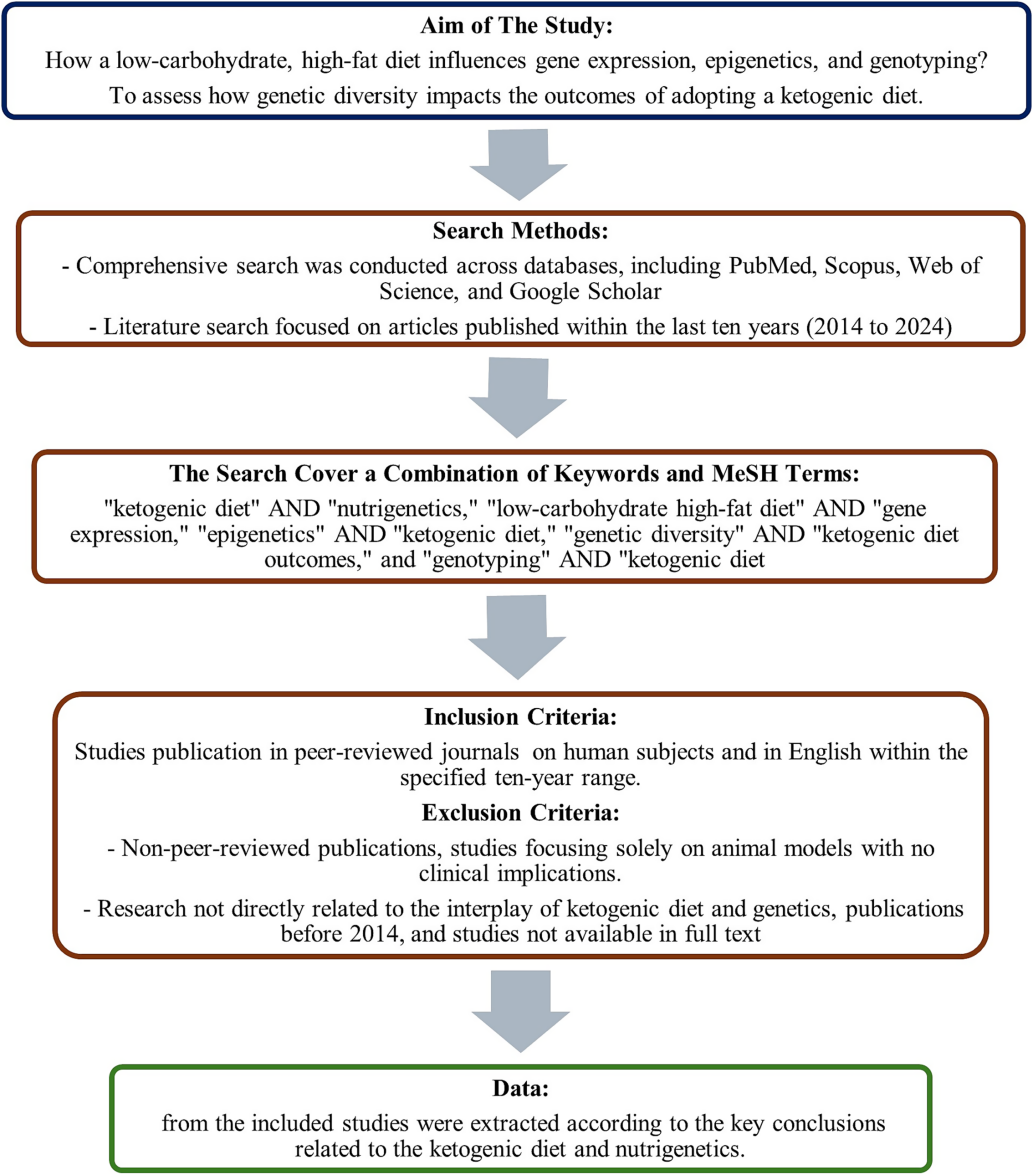
The diagram presents a summary of the methods of search conducted in the study’s database.

The selection process involved composing all articles, removing duplicates, and screening titles and abstracts by two independent authors for relevance to the review’s objectives. Full texts of potentially relevant studies were subsequently assessed for eligibility based on the inclusion and exclusion criteria. Data from the included studies were extracted according to the key conclusions related to the ketogenic diet and nutrigenetics. A flowchart of the process is presented in Figure 2.

This scoping review was conducted in accordance with the PRISMA-ScR (Preferred Reporting Items for Systematic Reviews and Meta-Analyses Extension for Scoping Reviews) guidelines (18). A standardized data charting form was used to extract key variables from each study, including study design, population characteristics, interventions or exposures, outcomes, and major findings. Two authors independently performed data extraction, with discrepancies resolved. The findings were synthesized using narrative description and thematic categorization to highlight key trends and research gaps. As this review involved the analysis of previously published literature without collecting or analyzing individual patient data, it was exempt from obtaining ethical approval in accordance with institutional regulations.

## 3 Nutrigenetics and nutrition-related genes

Nutrigenetics is a field that examines how variations in the human genome impact individual responses to nutrients, thereby influencing health outcomes (7). By examining genetic elements, including mutations, single nucleotide polymorphisms (SNPs), and epigenetic variations, nutrigenetics aims to elucidate the metabolic effects of nutrients and their contribution to overall health and disease predisposition (19). This section presents examples illustrating how alterations in nutrition-related genes can impact health status.

The *LCT* (lactase) gene encodes the lactase enzyme, which is essential for the digestion of lactose. Lactase expression typically decreases after weaning, leading to reduced lactose digestion in adulthood. However, individuals with a specific genetic variant in the *LCT* gene [lactase persistence allele, particularly the −13,910\u00B0C > T variant (rs4988235)] continue to produce lactase, allowing for lactose digestion beyond childhood. Individuals without this variant typically experience lactose intolerance (20). This genetic characteristic highlights the nutrigenetic connection between genetic makeup and dietary tolerances, as this trait is most commonly observed in populations with a historical reliance on dairy farming and milk consumption, such as Northern and Western Europeans, where lactase persistence can exceed 90% (20).

The metabolic effects of fructose and its genetic influences provide insights into the risk of metabolic disorders with implications that vary across populations. Two isoforms of fructokinase (ketohexokinase; KHK) are central to fructose metabolism: KHK-A and KHK-C. KHK-C is primarily found in the liver, kidneys, and intestines, playing a critical role in fructose-induced metabolic diseases. Genetically modified mouse models lacking both KHK isoforms are protected from metabolic problems associated with high-fructose diets, indicating that the issue stems from fructose metabolism, not fructose intake per se. In contrast, mice deficient only in KHK-A exhibit worsened metabolic effects, highlighting the essential role of KHK-C (21). Essential fructosuria, resulting from non-functional *KHK* mutations, causes elevated fructose levels in the urine but no adverse health effects, suggesting that KHK inhibition could be a therapeutic strategy. In humans, SNPs in the *KHK* gene, such as rs2304681 (Gly40Arg), have been linked to altered enzymatic activity and are associated with essential fructosuria (22).

Aldolase B (*ALDOB*) deficiency leads to hereditary fructose intolerance (HFI, rs1800546 [A149P]), with additional variants such as rs76917243 and rs78340951 also identified. They are characterized by adverse reactions to fructose in affected individuals (7). *ALDOB*-deficient mice exhibit signs of hepatic steatosis even without fructose consumption, suggesting a disturbance in the metabolism of endogenously produced fructose. These mutations are rare in East Asian and African groups (7, 22).

Furthermore, fructose metabolism may activate transcription factors such as carbohydrate-responsive element-binding protein (*ChREBP*, encoded by *MLXIPL*) and sterol-regulatory element-binding protein 1c (*SREBP1c*, encoded by *SREBF1*), which regulate lipid synthesis. Genetic polymorphisms in these transcription factors, such as rs3812316 in *MLXIPL* and rs11868035 in *SREBF1*, have been associated with altered lipid profiles and an increased susceptibility to metabolic syndrome (23). These interactions highlight the need for further investigation into how specific genes influence fructose metabolism and the associated risk of metabolic disorders.

Variations in the *FADS1* (fatty acid desaturase 1) gene influence the efficiency of converting plant-derived omega-3 fatty acids into more biologically active forms, which can impact cardiovascular health (24). The most studied SNP in *FADS1* is rs174546, where the derived T allele is associated with reduced desaturase activity. Allele frequencies vary widely among populations: the T allele is more common in East Asian and African populations, which may minimize conversion efficiency, whereas the ancestral C allele is more prevalent in European populations, correlating with higher desaturase activity and increased endogenous production of EPA and DHA (25).

Additionally, the *TAS2R38* (taste receptor) gene variant affects the ability to perceive certain bitter compounds, influencing dietary choices and potentially altering nutrient intake (26). The common SNP rs713598 (A49P) defines the “taster” and “non-taster” phenotypes. The “taster” variant (PAV haplotype) is more prevalent in African and South Asian populations, while the “non-taster” variant (AVI haplotype) is more frequently found in European populations (27).

The relationship between genetics and nutrition extends to protein metabolism. Sarcopenia, characterized by an age-associated decline in muscle mass and function, is influenced by genetic factors that control muscle physiology and its response to dietary protein (28). Vitamin D, in its active form (1α,25-dihydroxyvitamin D3), interacts with the vitamin D receptor (*VDR*) to influence the expression of numerous genes involved in immunity, bone health, and potentially protein metabolism and synthesis (29). Genetic variations in the *VDR* gene can influence an individual’s responsiveness to vitamin D. One well-studied single nucleotide polymorphism (SNP), rs2228570 (FokI), affects the start codon and the functional length of the VDR protein. The T allele (f variant) results in a less efficient VDR and is more prevalent in populations of Asian and African descent. In contrast, the C allele (F variant), associated with greater receptor activity, is more common in Europeans (30, 31).

Nutritional interventions, such as optimized protein intake and regular exercise, are crucial for maintaining musculoskeletal health. However, their effectiveness may be moderated by genetic factors, such as variations in the peroxisome proliferator-activated receptor *α* (*PPAR-α*), sterol regulatory element-binding protein 1 (*SREBP-1*), and acetyl-CoA carboxylase (*ACC*) genes, which are pivotal in muscle lipid metabolism and are influenced by the aging process (32).

The SNP rs1800206 (Leu162Val) in *PPAR-α* affects lipid metabolism and has been associated with variations in muscle energy utilization. The G allele (Val variant), which may enhance fat oxidation, is more common in African and South Asian populations (33). In contrast, the A allele (Leu variant) predominates in Europeans. In *SREBP-1*, the SNP rs11868035 has been linked to altered lipid profiles and insulin sensitivity, with certain risk alleles being more prevalent in East Asian and Hispanic populations (34). Similarly, the SNP rs2229416 in *ACC* has been associated with lipid accumulation in tissues and variable responses to dietary fat and physical activity. The T allele, which is more prevalent in European populations, is associated with increased lipogenesis, potentially influencing muscle lipid handling and metabolic health, particularly during aging (35).

Genetic variations can also have implications for lipid metabolism, influencing cholesterol levels and fatty acid composition. For example, polymorphisms in the apolipoprotein E (*APOE*) gene can alter lipid profiles. Polymorphisms in the *APOE* gene, particularly the SNPs rs429358 and rs7412, define the three major isoforms: ε2, ε3, and ε4. These alleles combine to form genotypes such as ε3/ε3 (neutral), ε2/ε3 (generally protective), and ε4/ε4 (associated with elevated LDL cholesterol and increased risk of cardiovascular disease) (36). The distribution of *APOE* alleles varies by ethnicity, with the cholesterol-raising ε4 allele more common in Northern Europeans and some African populations (15–20%) and less frequent in East Asians and Native Americans (<10%). The potentially protective ε2 allele is more prevalent in East Asian and European populations but rarer in African populations (37).

Individual variability can significantly impact metabolic processes, including those of carbohydrates, proteins, and fats. Specific genotypes may predispose individuals to metabolic disorders when consuming a diet high in specific types of sugars. For example, a diet rich in fructose has been correlated with an increased risk of developing metabolic-associated fatty liver disease (MAFLD) and enhanced lipogenesis (38).

These examples illustrate the implications of genetic variations on nutrient metabolism and nutritional habits, substantiating the principle of nutrigenetics in explaining the genetic determinants of nutritional responses and their impact on health and disease.

Personalized nutrition relies on the science of nutrigenetics, recognizing that individual responses to diet vary due to unique genetic compositions (39, 40). Understanding these nutrient-gene interactions is at the core of nutrigenetics and highlights the concept of individual variability. This variability, determined by genetic disposition, influences an individual’s susceptibility to certain diseases and overall health status. The goal of nutrigenetics is to use this information to develop personalized nutrition strategies that can optimize health and prevent disease based on an individual’s genetic background (41). Nutrigenetics provides a framework for personalized nutrition by examining the relationship between nutrition-related genes and their corresponding expression patterns.

## 4 Background of the low-carbohydrate-ketogenic diet

The low-carbohydrate ketogenic diet (LCKD) is characterized by a significant reduction in carbohydrate consumption coupled with increased fat intake to induce the production of ketone bodies (nutritional ketosis) (42). These ketones serve as an alternative primary energy source, particularly for the brain, in place of glucose. Under standard dietary conditions, carbohydrates are catabolized into glucose, triggering insulin release and facilitating glucose uptake into cells for energy utilization. With LCKD, the body initially utilizes glycogen reserves when carbohydrate intake is restricted. Once blood glucose levels are sufficiently reduced, the body activates gluconeogenesis, synthesizing glucose from non-carbohydrate substrates. If gluconeogenesis cannot adequately meet energy demands, the liver initiates ketogenesis, converting fatty acids into ketone bodies (43, 44).

Historically, the ketogenic diet was developed in the 1920s for the treatment of epilepsy and has demonstrated efficacy in reducing seizure frequency and intensity, particularly in patients who are unresponsive to conventional antiepileptic drugs (45). Beyond neurological applications, the LCKD has gained recognition as a dietary intervention for weight loss and the management of metabolic disorders, including obesity, type 2 diabetes, and metabolic syndrome. Its effectiveness for weight reduction is attributed to increased satiety from elevated fat and protein intake, reduced levels of hunger hormones, and an increase in energy expenditure due to the body’s metabolic adaptations to gluconeogenesis and ketogenesis (46). Furthermore, it can improve insulin sensitivity and glycemic control by attenuating postprandial blood glucose and insulin excursions, benefiting individuals with insulin resistance (47).

The impact of the LCKD on cardiovascular health remains an area of ongoing investigation. However, some studies suggest that LCKD may improve certain cardiovascular risk factors, such as reducing triglyceride levels and increasing high-density lipoprotein (HDL) cholesterol levels, despite the increased fat intake (48). Nevertheless, LCKD has potential limitations, including the risk of nutrient deficiencies and an increased metabolic burden for individuals with pre-existing health conditions (49).

The LCKD represents a substantial shift from glucose-based metabolism to the utilization of fat as the principal energy source. The therapeutic potential of this metabolic adaptation is evident in various clinical contexts, including the treatment of epilepsy, weight management, and the improvement of metabolic health (50).

As research continues, our understanding of how LCKD influences physiological functions and overall health will likely expand, potentially broadening its applicability in medical practice.

## 5 Low-carbohydrate-ketogenic diet/genes interactions

The LCKD exerts a profound influence on the body’s metabolic processes, extending to genetic and epigenetic alterations. This interaction between the diet and the genome is bidirectional: LCKD can modulate gene expression and epigenetic architecture, and an individual’s genetic makeup can influence their response to the diet (51).

The interplay between genetics and diet, with a specific focus on nutrigenetics and nutrigenomics, provides a critical framework for understanding the individualized effects of the ketogenic diet (52). Nutrigenetics examines how genetic variations influence an individual’s response to specific nutrients or dietary patterns, while nutrigenomics investigates how dietary factors can modulate gene expression and other molecular processes. Understanding these genetic and genomic factors can inform personalized dietary recommendations, optimizing the effectiveness of the ketogenic diet and minimizing potential risks based on individual genetic profiles.

In the following sections, we will explore the potential effects of LCKD on gene expression, epigenetic modifications, and gene polymorphisms. This analysis aims to provide a foundation for transforming the ketogenic diet from a general dietary approach for conditions such as obesity into a personalized nutritional plan that maximizes therapeutic potential while ensuring safety and efficacy tailored to individual needs. Further research is needed to fully elucidate the complex interactions between the ketogenic diet and the genome and to develop evidence-based personalized nutrition strategies.

### 5.1 The interplay between the ketogenic diet and gene polymorphism/genotyping

The interaction between diet and genes is a critical area of study in nutrigenomics, exploring how food and nutrition influence gene expression (53). Genotyping, the process of determining differences in genetic makeup by examining an individual’s DNA sequence, plays a crucial role in understanding how individuals respond to specific diets and their impact on health outcomes. Emerging research suggests that the low-carbohydrate ketogenic diet (LCKD) may exert a significant influence at the genetic level, particularly concerning gene polymorphisms (54).

The low-carbohydrate ketogenic diet (LCKD) highlights the relationship between gene polymorphisms and dietary responses, prompting considerations of individual genetic variations that significantly influence metabolic outcomes (6). For example, the fat mass and obesity-associated (*FTO*) gene plays a critical role in determining an individual’s fat metabolism, a vital aspect of LCKD’s functionality (55). Specific variants of the *FTO* gene have been associated with varying degrees of weight loss success on ketogenic diets.

The influence of LCKD on gene polymorphisms underscores the potential for personalized nutrition strategies based on genetic composition. Understanding an individual’s genotype can help tailor dietary recommendations to optimize health outcomes and prevent disease (56). For example, individuals with specific genetic variants that affect fat metabolism may experience greater benefits from the LCKD in terms of weight loss and improved metabolic health (6). However, it is essential to recognize that the effects of genetic variants on LCKD outcomes can be complex and are influenced by other factors, including lifestyle, environment, and additional genes.

While the effects of the ketogenic diet on various health conditions have been extensively investigated, the role of genetic polymorphisms in determining an individual’s response to the LCKD is gaining increasing attention. Examples of genetic variants and polymorphisms that are affected by or affect the low-carbohydrate ketogenic diet are presented in Table 1.

**Table 1:** The interplay between genetic variants, polymorphisms, and the low-carbohydrate ketogenic diet.

Category	Gene/pathway	SNP (rsID)	Population/ethnic group	Study type	% LCKD macros (Fat: Protein: Carbs)	Duration of LCKD	Human/animal	Description	Reference
Lipid metabolism	*APOE*	rs429358, rs7412	Northern Europeans, Africans	Human, RCT & Cohort	70:20:10 or 75:20:5	4–12 weeks	Human	*APOE* ε4 allele linked to elevated LDL-C response on LCKD	(119, 120)
	*FTO*	rs9939609	Europeans, South Asians, Africans	Human (Observational)	65–75:20–25:5–10	8–12 weeks	Human	A allele linked to reduced satiety and altered fat preference, influencing LCKD adherence and outcomes	(121)
Glucose metabolism	*PPARα*	rs1800206 (Leu162Val)	South Asians, Europeans	Human (Genotype-based diet response study)	70:20:10	6 weeks–3 months	Human	Variant linked to enhanced fatty acid oxidation and better adaptation to LCKD	(122)
	*SLC2A2*	rs5400	Europeans	Human (Cohort)	60–70:25:5–10	8 weeks	Human	Influences glucose transport; LCKD may improve glycemic control in variant carriers.	(123)
Neurological responses	*SCN1A/SCN2A*	Various (e.g., SCN1A rs121918779)	Global epilepsy cohorts	Human (Case series, trials)	Classic KD: 90:8:2	>6 months	Human	Variants respond well to LCKD through reduced neuronal excitability	(124)
Inflammatory pathways	*IL-6*	rs1800795 (−174G > C)	Europeans, Asians	Human (Intervention)	70:20:10	6–12 weeks	Human	C allele carriers show a greater inflammatory reduction on LCKD	(90)
	*NLRP3*	rs35829419 (Q705K)	European	Human & Animal	75:20:5	4–8 weeks	Both	Variant enhances inflammasome activity; LCKD attenuates this	(125)
Mitochondrial genetics	*mtDNA Haplogroups*	e.g., Haplogroup H, J	Europeans, Asians	Observational cohort	70:25:5	Not fixed	Human	Influence mitochondrial efficiency and ketone handling; haplogroups may predict response to LCKD.	(126)
	*UCP2*	rs659366	Europeans, Asians	Human (Intervention)	70:20:10	6–12 weeks	Human	Variant affects mitochondrial uncoupling efficiency, influencing LCKD-induced fat oxidation.	(127)
Appetite and satiety	*MC4R*	rs17782313	European, South Asian	Human (Genetic association)	65–75:20–25:5–10	8–12 weeks	Human	Linked to appetite regulation, the LCKD may suppress appetite in individuals carrying the risk allele.	(128)
	*LEPR*	rs1137101 (Q223R)	African, European	Human (Intervention & cohort)	70:20:10	4–12 weeks	Human	Influences leptin sensitivity; LCKD may stabilize appetite and insulin signaling.	(129)
Cardiovascular risks	*LDLR*	rs688, rs5925	Europeans, Middle Eastern	Human (FH cohorts, case–control)	70:20:10	Variable (6 + weeks)	Human	LDLR variants (especially in FH) can cause LDL-C spikes on LCKD	(130)
	*PCSK9*	rs11591147 (R46L)	European, African American	Human (GWAS & intervention)	75:20:5	8 weeks	Human	The gain-of-function variant impairs LDL clearance and may worsen lipid profiles on the LCKD.	(131)

### 5.2 The interplay between the low-carbohydrates ketogenic diet and epigenetic changes

The LCKD can induce epigenetic modifications that modulate gene expression in response to the altered metabolic state (57, 58). For example, research suggests that the LCKD can induce hypomethylation of the brain-derived neurotrophic factor (*BDNF*) gene, a critical regulator of neuroplasticity and cognitive function, which may contribute to the diet’s neuroprotective effects (59). A study on epileptic rats subjected to LCKD therapy demonstrated amelioration of DNA methylation-mediated gene expression changes by increasing adenosine levels (60, 61). Furthermore, the role of LCKD in aging has been linked to the positive regulation of epigenetic modifications, including nuclear lamin architecture, reduced telomere length, DNA methylation patterns, and chromatin structure (62).

Histones, the proteins around which DNA is coiled, are subject to chemical modifications that significantly influence gene expression. LCKD interventions have been observed to impact genes related to energy metabolism and storage (57). Specifically, an increase in histone acetylation levels within brain tissue, a modification associated with increased gene expression, has been documented. This elevation in histone acetylation could potentially enhance gene expression in neuroprotective processes, contributing to the diet’s effectiveness in mitigating epileptic seizures (14) and offering potential benefits in neurodegenerative disorders such as Alzheimer’s disease (63).

Histone deacetylase enzymes (HDACs) catalyze the removal of acetyl groups from lysine residues on histones and non-histone proteins. The 18 known HDAC enzymes are classified into four categories: Class I (Rpd3-like), Class II (Hda1-like), Class III (Sirtuins), and Class IV. LCKD and ketone bodies have been shown to suppress *HDAC* gene expression in human tumors, potentially impacting tumorigenesis (64). Beta-hydroxybutyrate, the predominant ketone body, has been reported to induce the enzymatic activities of sirtuins and upregulate the expression of mitochondrial respiratory chain enzymes in murine hippocampal neurons (13, 65).

LCKD can also affect the expression of non-coding RNAs (*ncRNAs*), including microRNAs (*miRNAs*) and long non-coding RNAs (*lncRNAs*), which regulate gene expression without being translated into proteins (64). The LCKD can regulate the expression of various non-coding RNAs (ncRNAs), which may impact disease pathways. For example, specific miRNAs that are dysregulated in cancer are normalized by LCKD, suggesting a potential anti-cancer effect through epigenetic regulation at the level of non-coding RNAs (ncRNAs). Consequently, LCKD has been applied in animal and human models as an adjuvant cancer treatment (64). Preclinical trials have demonstrated the impact of LCKD on reducing tumor development and improving survival in animal models of malignant glioma by modulating miRNAs, with similar effects observed in prostate cancer, colon cancer, and gastrointestinal carcinoma (66–68). Additionally, LCKD increases the expression of several miRNAs with tumor suppressor properties in glioma (69, 70).

In summary, the epigenetic modifications induced by the ketogenic diet can significantly impact health and disease, potentially mediated through altered gene expression of anti-inflammatory and neuroprotective factors. Consequently, the ketogenic diet offers therapeutic potential beyond epilepsy, including metabolic disorders, neurodegenerative diseases, and cancer. The modulation of epigenetic markers also underscores the potential for personalized nutrition strategies based on individual epigenetic profiles, opening new avenues for tailored dietary interventions (71).

**Figure 3:**
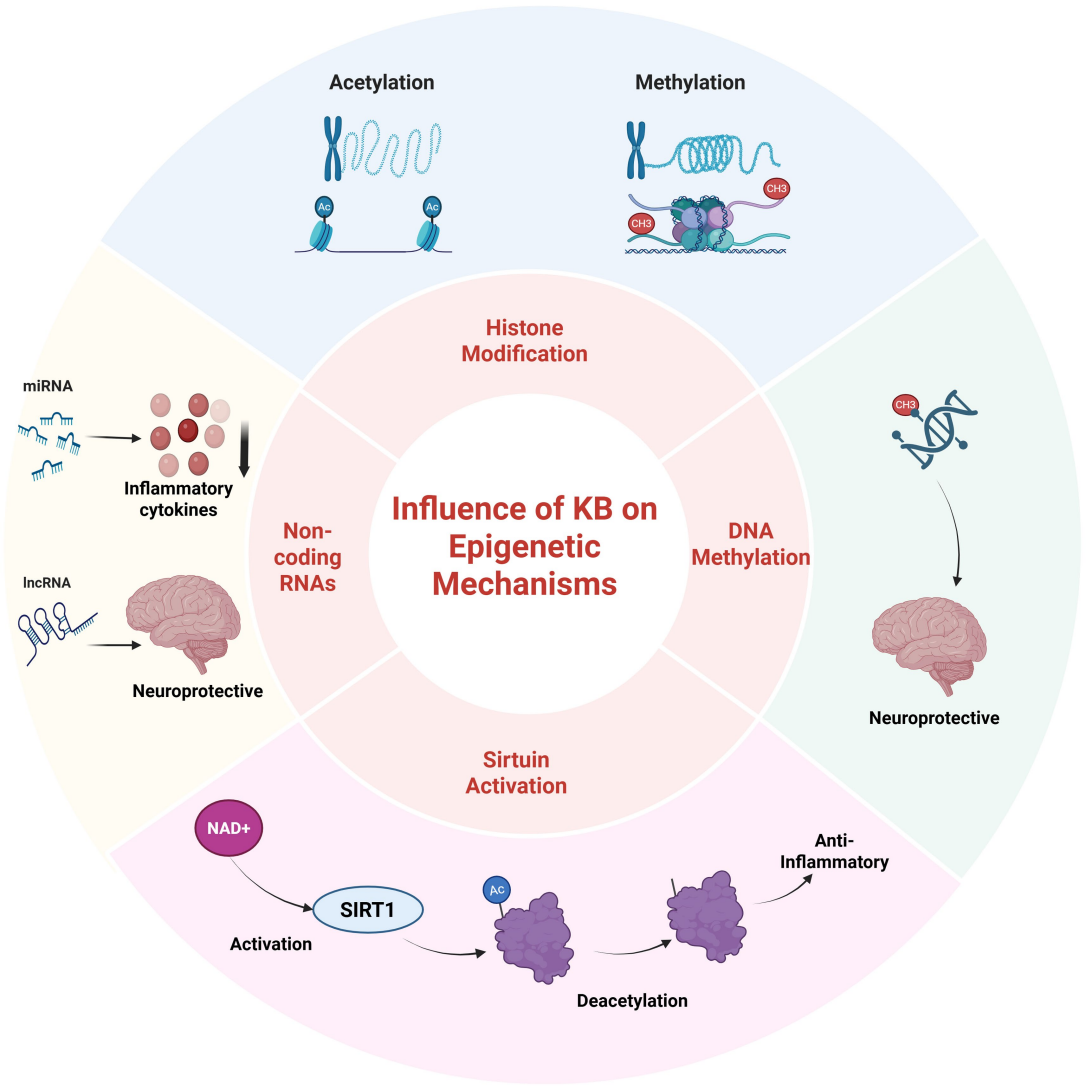
The influence of ketone bodies on the key epigenetic regulatory mechanisms. Created with BioRender.com Elsamanoudy, https://BioRender.com/8cog64y.

Examples of genes affected by the low-carbohydrate ketogenic diet at the epigenetic level are presented in Table 2 and Figure 3.

**Table 2:** Examples of genes affected by the low-carbohydrate ketogenic diet’s epigenetic modifications.

Epigenetic modification	Impact on gene expression	Examples of affected genes	Reference
Histone modifications	Alters histone acetylation and methylation, enhancing gene expression for anti-inflammation and neuroprotection.	Anti-inflammatory genes (e.g., I*L-10, TGF-β, Nrf2, HO-1*), neuroprotective genes (e.g., *CREB, SYP*)	(132)
DNA methylation	Changes in dietary fat impact methyl donor availability, leading to altered DNA methylation and expression of genes for inflammation and neuronal function.	Neuroprotective genes (e.g., *BDNF, NGF, FGF2*), genes affecting synaptic plasticity (e.g., *SYN1*)	(133)
Non-coding RNAs	It affects the expression of microRNAs and long non-coding RNAs, regulating post-transcriptional gene expression in inflammation and neuroprotection.	miRNAs regulating inflammatory cytokines (e.g., *miR-155, miR-146a*), lncRNAs involved in neuroprotection and inflammation control	(134)
Sirtuin activation	Increases NAD + levels, activating *SIRT1* and other sirtuins, which deacetylate proteins to promote anti-inflammatory and neuroprotective effects.	Genes related to aging and metabolism (e.g., *FOXO3, PGC-1α, AMPK, SIRT3*)	(13, 135)

### 5.3 The interplay between the low-carbohydrate ketogenic diet and genes at the transcriptional and translational levels

The LCKD induces a fundamental shift in energy metabolism, transitioning the body from glucose utilization to the conversion of fats into ketone bodies (72). This metabolic adaptation elicits a cascade of cellular responses, including alterations in gene expression profiles (71). LCKD interventions have been shown to modulate the expression of genes involved in metabolic pathways, inflammatory responses, and cellular stress responses (49).

A key mechanism by which the LCKD influences gene expression involves the modulation of signaling pathways that regulate gene transcription (73). The diet can affect the activity of specific kinases and transcription factors that govern cellular and mitochondrial transcription and translation, thereby influencing gene expression (74). For example, the activation of AMP-activated protein kinase (AMPK), a critical cellular energy sensor, has been observed in response to LCKD interventions. AMPK activation can influence the expression of genes involved in energy metabolism, promoting fatty acid oxidation and inhibiting lipid synthesis, consistent with the metabolic demands of the KD (75).

The LCKD can upregulate genes such as the peroxisome proliferator-activated receptor alpha (*PPARα*), a key regulator of fat metabolism at both the transcriptional and translational levels. This effect enhances the body’s capacity to utilize fat as an energy source, reflecting the underlying mechanisms of the diet (76, 77). *PPARα* activation promotes the transcription of genes involved in fatty acid uptake, beta-oxidation, and ketone body synthesis.

Furthermore, the LCKD can alter mitochondrial gene expression and enhance mitochondrial bioenergetics (74). Ketone bodies can enhance mitochondrial function in neurological disorders, including Parkinson’s disease, Alzheimer’s disease, amyotrophic lateral sclerosis, epilepsy, and neurotrauma, by increasing the expression of antioxidant enzymes. This helps to mitigate oxidative stress-induced mitochondrial damage and enhance mitochondrial function (78–80). However, the specific effects of LCKD on mitochondrial function and gene expression can vary depending on the tissue type, the duration of the intervention, and individual factors.

Specific genes involved in ketone body metabolism that are influenced by the LCKD include succinyl-CoA:3-ketoacid CoA transferase (*SCOT*) and 3-hydroxy-3-methylglutaryl-CoA synthase 2 (mitochondrial; *HMGCS2*) (81). *SCOT*, which is essential for the utilization of ketone bodies for energy, has been shown to exhibit reduced expression in the heart and skeletal muscle following ketone diet (KD) consumption, regardless of the diet’s duration. Conversely, *HMGCS2*, which is critical for ketogenesis, has been shown to exhibit increased expression in the hearts of mice on a LCKD (82). This pattern, including decreased SCOT expression and increased *HMGCS2* expression, has also been observed in the hearts of mice on a ketogenic diet for 5 weeks, as documented by (83). These changes in gene expression reflect the metabolic adaptations that occur in response to the LCKD (83).

### 5.4 The alteration in gut microbiota by low-carbohydrates ketogenic diet and its impact on gene modification

The human gut microbiota, a complex community of microorganisms, plays a crucial role in maintaining host health and influencing the pathogenesis of diseases. The composition of this microbiota is significantly influenced by dietary factors, with alterations in diet leading to measurable shifts in the microbial ecosystem (84).

The LCKD, characterized by a substantial reduction in carbohydrate intake and an increase in fat consumption, has been shown to induce alterations in the gut microbiota composition (85). Studies have reported that LCKD interventions can increase the relative abundance of certain bacterial taxa, such as *Akkermansia Muciniphila* and *Lactobacillus* (86), while decreasing the relative abundance of others, such as certain members of the Firmicutes phylum (87). These changes in microbiota composition have been associated with various health benefits, including improved metabolic profiles, reduced systemic inflammation, and enhanced gut barrier function (12, 88). However, the specific changes in gut microbiota composition and their functional consequences can vary depending on individual factors, dietary composition, and the duration of the LCKD intervention.

The gut microbiota can influence host gene expression through various mechanisms, including the production of short-chain fatty acids (SCFAs) such as butyrate, propionate, and acetate. Gut bacteria produce these SCFAs during the fermentation of dietary fibers (89). While some studies suggest that LCKD interventions can support SCFA production (90), the overall impact of LCKD on SCFA production and gut fermentation patterns remains an area of active investigation. SCFAs can exert epigenetic effects, such as the inhibition of histone deacetylases (HDACs) (91). HDAC inhibition can lead to alterations in gene expression, potentially promoting anti-inflammatory responses and enhancing intestinal barrier function (92). Additionally, LCKD-induced alterations in gut microbiota composition can influence the metabolism of bile acids, which act as signaling molecules that affect gene expression related to metabolism and inflammation via the farnesoid X receptor (*FXR*) (93) and the G protein-coupled bile acid receptor 1 (*GPBAR1*) (94).

The interplay between the ketogenic diet, gut microbiota, and gene modification holds potential therapeutic implications. For example, the anti-inflammatory effects resulting from alterations in the microbiota and subsequent gene modifications may have beneficial effects in conditions associated with chronic inflammation, such as inflammatory bowel disease (IBD) (95), obesity, and type 2 diabetes (96). Furthermore, the effects of the LCKD on the gut-brain axis, potentially mediated through microbiota-induced epigenetic changes, suggest possible benefits for neurological conditions, including epilepsy, Alzheimer’s disease, and autism spectrum disorders (97). However, the evidence supporting these therapeutic applications remains preliminary, and further research is needed to elucidate the mechanisms of action fully and to determine the long-term efficacy and safety of LCKD interventions for these conditions.

### 5.5 The impact of gene diversity on the outcome of the low-carbohydrate ketogenic diet

Genetic diversity represents a significant factor influencing the variability observed in LCKD outcomes. Interindividual differences in genetic makeup can substantially impact the efficacy and safety of the diet. Genetic variations affecting lipid metabolism, energy expenditure, and appetite regulation can modulate nutrient metabolism and individual responses to LCKD interventions (49).

While some individuals may experience improvements in weight management and metabolic health, others may exhibit minimal benefits or adverse reactions, reflecting their unique genetic predispositions. For example, polymorphisms in the apolipoprotein A2 (*APOA2*) gene, which plays a role in lipid metabolism, have been associated with variations in satiety and weight loss outcomes in response to high-fat diets (98, 99). The fat mass and obesity-associated (*FTO*) gene, implicated in energy expenditure and dietary preferences, may also influence the effectiveness of the LCKD. Specific variants of the *FTO* gene have been linked to reduced metabolic rate, potentially attenuating the weight loss effects of the LCKD (100). Further research is needed to fully elucidate the mechanisms by which these genetic variants influence LCKD outcomes.

The SNP rs9939609 (T/A) in intron 1 of the *FTO* gene is one of the most extensively studied genetic variants associated with obesity. Individuals who carry the A allele (the risk variant) tend to experience reduced satiety, a preference for energy-dense, high-fat foods, and a higher total energy intake. This preference may initially support better adherence to high-fat diets such as the low-carbohydrate ketogenic diet (LCKD) (101). According to Doaei et al. (2019), the *FTO* gene significantly influences both the short-term and long-term effects of low-carbohydrate ketogenic diets (LCKD) (102). Individuals with risk alleles, such as rs9939609 (T/A) and rs9930506 (G/A), tend to have elevated *FTO* expression, which promotes glycolysis and fat accumulation. The A allele of both SNPs is associated with increased body mass index (BMI), appetite dysregulation, and a greater risk of obesity. They are also associated with elevated *FTO* gene expression and a higher tendency for fat accumulation, as well as a greater risk of obesity. In the short term, LCKD may reduce FTO expression by lowering glucose and insulin levels, improving weight loss and metabolic control in these individuals. However, in the long term, persistent FTO overexpression may reduce the resting metabolic rate and increase appetite, potentially diminishing the effectiveness of the LCKD (102).

Additionally, *FTO* may enhance cancer cell proliferation via the PI3K/AKT pathway, making its modulation through LCKD particularly relevant in cancer-prone individuals; however, the outcome may depend on the genotype (102). Thus, while individuals with this *FTO* variant may respond favorably to LCKDs in the short term due to dietary preference, their metabolic profile may limit the sustained benefits (100). These findings suggest that the *FTO* genotype can influence both behavioral and physiological responses to ketogenic dietary interventions.

Genetic variations also significantly influence an individual’s ability to process dietary fats, a primary component of LCKDs (103). For instance, variations in the *APOE* gene can modulate an individual’s ability to tolerate and metabolize different types of fats. Individuals carrying the A*POE4* allele may exhibit an increased risk of hypercholesterolemia and cardiovascular disease when consuming a diet rich in saturated fats, a common characteristic of many LCKDs (104). Therefore, the LCKD may not be universally suitable, particularly for individuals with a pre-existing risk of cardiovascular disease (49). It is important to note that the impact of *APOE4* on cardiovascular risk in the context of LCKD is an area of ongoing investigation.

Beyond metabolic and fat tolerance considerations, genetic diversity can influence the risk of adverse health outcomes associated with LCKD implementation (105). Individuals with specific genetic mutations may be at increased risk for developing non-alcoholic fatty liver disease (NAFLD) when consuming a high-fat diet. For example, the I148M variant of the patatin-like phospholipase domain-containing protein 3 (*PNPLA3*) gene has been associated with increased hepatic fat accumulation, predisposing carriers of this variant to a higher risk of non-alcoholic fatty liver disease (NAFLD) when consuming a high-fat diet (106). Similarly, mutations in the transmembrane 6 superfamily member 2 (*TM6SF2*) gene can lead to reduced secretion of very low-density lipoprotein (VLDL), resulting in hepatic fat accumulation and an increased risk of non-alcoholic fatty liver disease (NAFLD) (107). Furthermore, variants in the membrane-bound O-acyltransferase domain-containing 7 (*MBOAT7*) gene have been linked to an increased risk of hepatic fat accumulation and inflammation, key features of NAFLD (108). These findings underscore the importance of considering genetic risk factors in the context of LCKD interventions.

Responses to dietary interventions such as the ketogenic diet may vary across ethnic groups, underscoring the importance of considering ethnic diversity in personalized nutrition research and the need for population-specific dietary recommendations. The study by Oh et al. (2022) demonstrated significant ethnic-specific differences in the association between low-carbohydrate diets (LCDs) and mortality outcomes in a multiethnic U. S. cohort (109). Among Hispanic participants, moderate carbohydrate intake was associated with a significantly lower risk of all-cause and non-cardiovascular mortality, with hazard ratios ranging from 0.58 to 0.83 across the second to fifth quintiles of LCD scores compared to the lowest quintile. This protective association was not observed in non-Hispanic groups, including African American, Chinese American, and non-Hispanic White participants. A U-shaped relationship between carbohydrate intake and mortality was evident only in the Hispanic subgroup, suggesting a narrow optimal carbohydrate range for this population. The differential response may be attributed to unique dietary patterns and metabolic characteristics; Hispanic participants had higher intakes of total carbohydrates, protein, and fiber but also demonstrated lower insulin sensitivity, potentially increasing their vulnerability to the adverse effects of high-carbohydrate diets. These findings underscore the importance of personalized dietary guidelines that account for racial and ethnic variations in macronutrient metabolism and health outcomes (109).

## 6 Discussion

Nutrigenetics examines the influence of genetic variations on individual nutrient responses, which can impact dietary needs and susceptibility to diet-related diseases. Genetic variations, such as single nucleotide polymorphisms (SNPs), can impact the function of enzymes, transporters, and receptors involved in nutrient metabolism, absorption, and utilization, thereby altering the bioavailability and effectiveness of nutrients (110).

The LCKD has garnered attention for its therapeutic potential in weight management, diabetes control, and neurological disorders. However, individual responses to the LCKD vary considerably, and genetic differences may contribute to this variability. The LCKD induces a significant metabolic shift from carbohydrate to fat utilization, leading to substantial metabolic changes. While genetics can influence the efficacy and safety of a diet (49), the extent of this influence remains a subject of ongoing research.

The premise of nutrigenomics lies in its potential to personalize dietary recommendations based on individual genetic profiles, moving away from generalized advice (41). By analyzing an individual’s genetic variations, healthcare providers could potentially tailor the LCKD to maximize benefits and minimize risks. This might involve identifying genetic markers that predict individual responses to the diet and adjusting macronutrient ratios, supplementation, and dietary restrictions accordingly (111).

However, the application of nutrigenomics to personalize the LCKD faces significant challenges (112). The complexity of genetic interactions, the influence of environmental factors, and the need for robust and validated genetic tests all present significant obstacles. Furthermore, ethical considerations, including privacy, consent, and access to genetic information, require careful attention (112, 113).

While the intersection of nutrigenomics and the LCKD holds promise for transforming dietary management and personalized healthcare, several limitations must be addressed. The potential of genetic information to personalize nutritional recommendations enables clinicians to tailor the LCKD and other diets to optimize individual health outcomes in weight management, diabetes, cardiovascular disease, and neurological conditions.

By elucidating how genetic variations influence individual reactions to the LCKD, healthcare providers can more effectively utilize diet as a management tool in conditions such as epilepsy, type 2 diabetes, and obesity, potentially enhancing the diet’s therapeutic benefits while minimizing adverse effects. Nutrigenomics may enable the identification of individuals at increased risk of adverse reactions to certain diets based on their genetic profile, improving risk assessment and management strategies to ensure dietary recommendations do not inadvertently heighten health risks.

Integrating nutrigenetic testing into clinical practice may become necessary for effective dietary counseling and the management of diet-related diseases. Moreover, the development of affordable and accessible genetic tests, as well as the training of healthcare professionals in their interpretation and application, is also needed.

Further research is essential to validate the clinical benefits of nutrigenetically tailored diets. Physicians and nutritionists must stay informed about the latest research to effectively integrate nutrigenetics into practice and counsel patients with the most current insights into gene-diet interactions.

The integration of genetics with dietary interventions encounters notable obstacles. The complex interplay between genes and diet, where multiple genes may influence an individual’s reaction to a single nutrient (114), proposes difficulties in making precise dietary recommendations. Despite advancements in identifying genetic variants associated with nutrition and metabolism, a comprehensive understanding remains incomplete, with many variants yet to be discovered and the full implications of known ones yet to be understood (41). Moreover, individual variability in diet response, influenced by genetic and non-genetic factors such as age, sex, lifestyle, microbiome composition, and environmental conditions, complicates the customization of dietary advice (115–117).

The application of nutrigenomics in dietary guidance is also hindered by practical issues such as the cost and accessibility of genetic testing, which remains prohibitive for many, especially in resource-limited settings. The successful integration of nutrigenomics into healthcare requires professionals to possess specialized genetic knowledge, potentially necessitating mandatory training to accommodate personalized counseling as a standard practice (7). The lack of standardized, evidence-based guidelines for using genetic information in dietary planning further risks inconsistent or inappropriate application of nutrigenomics principles, underscoring the need for clear directives (112).

Several scientific and practical challenges impede the widespread implementation of nutrigenetics in LCKD interventions. These include the complexity of gene-diet interactions, the incomplete understanding of relevant genetic variants, and difficulty in predicting individual dietary responses (41). Practical obstacles include the accessibility and cost of gene testing, the need for specialized knowledge among healthcare professionals, and the absence of standardized, evidence-based guidelines for implementing nutrigenetic principles. The ethical considerations surrounding genetic testing and data privacy also warrant careful attention (118).

Addressing these challenges requires a concerted effort focused on (1) advancing rigorous scientific research to address existing knowledge gaps, including well-designed clinical trials; (2) improving the accessibility and affordability of genetic testing through technological advancements and cost-reduction strategies; and (3) developing clear, evidence-based guidelines for nutrigenetic applications, informed by expert consensus and clinical data. As the field evolves, ongoing monitoring of emerging evidence and adopting a holistic perspective that integrates genetic data with lifestyle and environmental factors will be crucial. While integrating nutrigenetics into clinical practice holds the potential for more personalized and effective healthcare, further research and careful consideration of the associated challenges are necessary to ensure its responsible and beneficial implementation in diet and disease management.

## 7 Conclusion

The interplay between LCKD and genetic factors represents a complex, bidirectional relationship. LCKD can modulate gene expression and epigenetic profiles, leading to measurable physiological effects. Conversely, an individual’s genetic constitution can influence their response to the diet, impacting both its efficacy and safety profile. A comprehensive understanding of these interactions is crucial for developing personalized dietary strategies that optimize the benefits of the LCKD while minimizing potential risks, thereby contributing to individualized nutritional guidance within a precision health framework.

Integrating nutrigenomic principles into dietary interventions, particularly with the LCKD, offers a promising avenue for personalized healthcare, potentially refining dietary management, disease prevention, and individualized treatment strategies. This approach aims to align dietary recommendations with individual genetic profiles, moving beyond generalized guidelines to improve health outcomes in weight management, diabetes, cardiovascular diseases, and neurological disorders. By further elucidating the intricate relationships between genetics and diet, healthcare practitioners may be able to optimize the therapeutic application of the LCKD, tailoring it to minimize risks and enhance benefits in specific conditions, including epilepsy, type 2 diabetes, and obesity. However, current evidence supporting these applications remains limited.
